# Infections Following Kidney Transplantation After Exposure to Immunosuppression for Treatment of Glomerulonephritis

**DOI:** 10.1053/j.ajkd.2023.10.016

**Published:** 2023-12-30

**Authors:** David Massicotte-Azarniouch, Randal K. Detwiler, Yichun Hu, Ronald J. Falk, Manish K. Saha, David van Duin, Susan L. Hogan, Vimal K. Derebail

**Affiliations:** UNC Kidney Center, Division of Nephrology and Hypertension, University of North Carolina at Chapel Hill, Chapel Hill, North Carolina; Division of Nephrology, Department of Medicine, University of Ottawa, Ontario, Canada; UNC Kidney Center, Division of Nephrology and Hypertension, University of North Carolina at Chapel Hill, Chapel Hill, North Carolina; UNC Kidney Center, Division of Nephrology and Hypertension, University of North Carolina at Chapel Hill, Chapel Hill, North Carolina; UNC Kidney Center, Division of Nephrology and Hypertension, University of North Carolina at Chapel Hill, Chapel Hill, North Carolina; UNC Kidney Center, Division of Nephrology and Hypertension, University of North Carolina at Chapel Hill, Chapel Hill, North Carolina; Division of Infectious Diseases, Department of Medicine, University of North Carolina at Chapel Hill, Chapel Hill, North Carolina; UNC Kidney Center, Division of Nephrology and Hypertension, University of North Carolina at Chapel Hill, Chapel Hill, North Carolina; UNC Kidney Center, Division of Nephrology and Hypertension, University of North Carolina at Chapel Hill, Chapel Hill, North Carolina

## Abstract

**Rationale & Objective:**

Kidney transplant patients with glomerulonephritis (GN) as their native disease commonly have received pretransplant immunosuppression (PTI). This may contribute to the immunosuppression burden potentially increasing the risk for infections after transplantation.

**Study Design:**

Single-center, retrospective cohort study.

**Setting & Participants:**

Recipients of a kidney transplant from January 2005 until May 2020 at a tertiary care university teaching hospital.

**Exposure:**

Patients with GN as their native kidney disease who received PTI for treatment of GN (n = 184) were compared with nondiabetic recipients of kidney transplants who did not receive PTI (n = 579).

**Outcome:**

First occurrence after transplantation of an infection outcome, either viral (BK or cytomegalovirus [CMV] infection) or bacterial.

**Analytical Approach:**

Cox regression analysis adjusted for age at transplant, sex, race, donor type, year of transplant surgery, dialysis vintage, receipt of T-cell depleting induction, and CMV transplant status.

**Results:**

Over a median follow-up period of 5.7 years, patients with GN PTI were not at an increased risk for developing any first viral infection compared with controls (adjusted HR [AHR] 0.69 [95% CI, 0.52–0.91]) nor at increased risk for specific viral infections: BK infection 19.6% vs 26.3% (AHR 0.72 [95% CI, 0.50–1.05]) or CMV infection, 24.5% vs 29.0% (AHR, 0.76 [95% CI, 0.54–1.07]), respectively. There was also no increased risk of developing a first bacterial infection: 54.5% vs 57.5% (AHR, 0.90 [95% CI, 0.71–1.13]). These findings of no increased risk for infection were independent of the type of PTI used (cyclophosphamide, rituximab, mycophenolate mofetil, or calcineurin inhibitor) or the type of T-cell depleting induction therapy (alemtuzumab or antithymocyte globulin) administered.

**Limitations:**

Single-center study, no data on methylprednisone use for PTI, unmeasured confounding.

**Conclusions:**

Use of PTI for the treatment of GN was not associated with an increased risk of viral (BK or CMV) or bacterial infection after transplantation. Additional surveillance for infection after transplantation for patients who received PTI may not be necessary.

## Graphical Abstarct

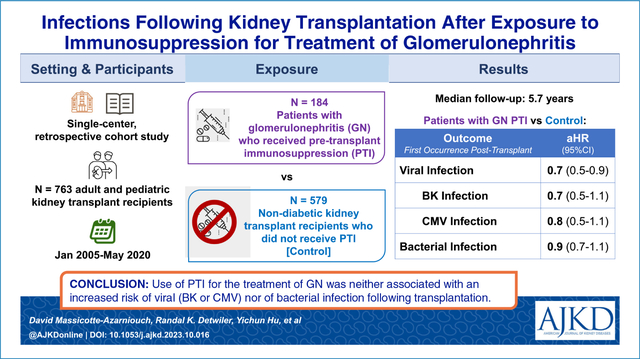

Kidney transplant patients are at high risk for infectious complications due to the need for life-long immunosuppression.^1–3^ Greater exposure to immunosuppression at the time of transplant and throughout the posttransplant course increases the risk for developing infections.^4,5^ Therefore, the overall immunosuppression burden seems to impact the risk for a transplant patient to develop infections. A potentially important but often overlooked contributor to a kidney transplant recipient’s immunosuppression burden is exposure to immunosuppression before the transplant.

Approximately 20% of kidney transplant recipients have glomerulonephritis (GN) as the cause of their end-stage kidney disease (ESKD).^6^ Many such patients will have received pretransplant immunosuppression (PTI) for treatment of their GN. They may be exposed to a multitude of different immunosuppressants, at significant doses, and for periods of time varying from months to years. This accrued immunosuppression exposure could leave a patient more vulnerable to developing complications after transplant. Studies have suggested it could increase the risk for certain types of malignancy after kidney transplant.^7–9^ Whether PTI for treatment of GN could also increase infection risk after kidney transplant remains unclear.

This study examined the risk of developing a viral or bacterial infection after kidney transplant in patients with GN as their native disease who received PTI for treatment of their glomerular disease. We hypothesized that GN PTI would be associated with an increased risk for both viral and bacterial infection after transplant.

## Methods

### Study Design, Setting, and Participants

We conducted a single-center, retrospective study at the University of North Carolina at Chapel Hill (UNC) of all kidney transplant recipients (pediatric and adult) at UNC Hospitals between January 1, 2005, and May 31, 2020. The study participants were identified through the solid-organ transplant registry at UNC where patients are followed throughout their transplant course. Kidney transplant recipients are seen at UNC at least twice a year, and outcomes are captured during those visits. The methods used for this study are similar to those in a published study by our group examining the risk for malignancy after transplant associated with the use of immunosuppression before transplant for treatment of GN.^10^ Local institutional review board ethics was obtained before commencing the study. Due to the retrospective nature of the study, informed consent was waived. This study was reported as per the STROBE guidelines for observational studies.

The exposure of interest was receipt of PTI in kidney transplant recipients with GN as their native disease (GN PTI), which was captured in the patient registry as required by the United Network for Organ Sharing and was confirmed and further specified (for patients with GN) by chart review. Study participants with GN PTI included those with focal segmental glomerulosclerosis (FSGS), IgA nephropathy (IgAN), lupus nephritis (LN), antineutrophil cytoplasmic antibody (ANCA) vasculitis, membranous nephropathy (MN), membranoproliferative glomerulonephritis (MPGN), cryoglobulinemia, C3 glomerulopathy, anti–glomerular basement membrane (GBM) disease, atypical hemolytic uremic syndrome, fibrillary GN, and immunotactoid GN. The doses of PTI for treatment of GN were obtained from chart review.

A study participant was classified as exposed to PTI if any of the following occurred before transplant for treatment of GN: receipt of at least 1 dose of intravenous (IV) cyclophosphamide or rituximab, or prescriptions provided for oral cyclophosphamide, mycophenolate (MMF), azathioprine, calcineurin inhibitor (CNI), or oral glucocorticoid of ≥20 mg prednisone equivalent per day for ≥4 weeks (we did not capture methylprednisolone pulses because these data were not readily available nor consistently recorded). We quantified PTI exposure for each patient and each drug group by ascertaining total grams of cyclophosphamide (IV and oral) and rituximab used and total months of MMF, azathioprine, and CNI use. We could not quantify the exact duration of exposure to high-dose prednisone because dosage and duration were not consistently captured in the medical record. We also did not capture information on rarer treatments for GN such as eculizumab, etanercept, or belimumab. Item S1 explains how we proceeded when the information on dosage or duration of PTI was incomplete.

Patients with GN who were exposed to PTI were compared with a control population who had ESKD due to (1) GN but were never exposed to PTI; (2) hypertension; (3) polycystic kidney disease (PKD); (3) congenital anomalies of the kidney and urinary tract (CAKUT); (4) Alport syndrome; and (5) any other cause of ESKD where the individual would not have received PTI or chemotherapy for treatment of that disease. We excluded kidney transplant recipients with diabetes as their designated native disease, those who had received a prior organ transplant, those who received a multiorgan transplant, and those who received chemotherapy or immunosuppression before transplant for indications other than treatment of GN. These exclusions were applied to ensure the exposure of interest was not contaminated by immunosuppression used for non-GN diseases and to obtain more similar exposure and control groups in terms of inherent risks for adverse outcomes.

### Covariates

Baseline variables were selected a priori based on availability in the patient chart and what was felt to be a potentially important confounder between exposure and outcome: age at transplant, sex, race, cause of ESKD, year of transplant, type of transplant (living donor, donation after brain death, donation after cardiac death), time on dialysis (“dialysis vintage,” including pre-emptive transplants where vintage was considered zero), Epstein-Barr virus (EBV) and cytomegalovirus (CMV) donor-recipient status, pretransplant panel reactive antibodies (PRA; the most recent value before transplant), type of induction therapy (T-cell depleting or not), type of T-cell depleting therapy (either alemtuzumab or antithymocyte globulin [ATG]), maintenance therapy initially prescribed after transplant (tacrolimus or other), presence of diabetes as a comorbidity (without being cause of ESKD, based on chart review), smoking history before transplant, and presence of delayed graft function immediately after transplant. Item S2 details the usual immunosuppression protocol at our institution during the study period.

### Outcomes

Study outcomes were (1) first occurrence of a viral infection (BK or CMV infection) and (2) first occurrence of a bacterial infection. BK virus infection was defined as either (1) BK DNA polymerase chain reaction (PCR) level ≥ institutional cutoff (quantifiable BK viremia) or (2) BK virus nephropathy confirmed on kidney biopsy. CMV infection was defined as either (1) CMV DNA PCR ≥ institutional cutoff (quantifiable CMV viremia) or (2) biopsy-confirmed CMV tissue invasive disease, regardless of whether there was CMV viremia. Bacterial infection was defined as one of the following: (1) urinary tract infection (urinalysis with positive nitrites and/or leukocytes, or a positive urine culture, with decision to treat with antibiotics); (2) pneumonia (chest X-ray demonstrating infiltrates with decision to treat with antibiotics); (3) bacteremia (any positive blood culture, regardless of organism); (4) *Clostridioides difficile* infection (any positive *Clostridioides difficile* stool toxin assay); (5) wound infection (positive wound culture with decision to treat with antibiotics); or (6) hospitalization at UNC Hospital for an infectious event not classified as any of the other infections (determined through medical record review).

At our center, CMV prophylaxis with valganciclovir (adjusted for kidney function) is given based on donor-recipient risk status. Monitoring for BK infection is performed using urine cytology decoy cell screening (see Item S2).

### Statistical Analysis

Descriptive statistics were calculated for each covariate (median with interquartile range and number with percentage, where appropriate) in both study groups with differences evaluated using Wilcoxon 2 sample test and Fisher’s exact test. *P* < 0.05 was considered statistically significant. The median (with IQR) grams of cyclophosphamide and rituximab received before transplant as well as the months of MMF, azathioprine, and CNI use were also calculated in both study groups.

Counts with percentages, cumulative incidence rates at 1, 2, and 5 years, and Kaplan-Meier survival curves were determined for each infection outcome. Hazard ratios (HR) with 95% confidence intervals for outcomes based on presence or absence of PTI were estimated using Cox proportional hazards models. Multivariable Cox regression models were used to generate adjusted hazard ratios (AHR), accounting for the following covariates: age at transplant, sex, race, donor type, year of transplant surgery, dialysis vintage, receipt of T-cell-depleting induction, and CMV transplant status. The selection of these covariates was to maintain an event-to-variable ratio of 5:10. The index date (time zero) for the start of the follow-up period was the date of kidney transplant. The end of the follow-up period was the earliest of these events: occurrence of an infection outcome, death, graft loss (permanent return to dialysis or retransplantation), loss to follow-up (transfer to another program and no longer followed at our center), or end of the study period (June 1, 2021, to allow a minimum 1 year of follow-up for each study participant). We also calculated AHRs for outcomes based on whether the PTI was cyclophosphamide, rituximab, MMF, or CNI. We did not perform these analyses for azathioprine due to the small number of patients who received azathioprine as PTI. All analyses and plots were conducted with SAS software (version 9.4; SAS Institute).

### Additional Analyses

We performed the following additional outcome analyses. First, we examined the risk associated with GN PTI for only viral infection and only bacterial infection; that is, a viral infection outcome was recorded when study participants developed a viral infection during their posttransplant course without ever developing a bacterial infection, and a bacterial infection outcome was recorded when a study participant developed a bacterial infection without ever developing a viral infection. Second, we examined the risk associated with PTI for viral and bacterial infection—that is, study participants who developed both a viral and a bacterial infection during their posttransplant course. Third, given the difference in lasting lymphocyte depletion seen between these agents,^11^ we looked at the risk associated with GN PTI for infections stratified by the type of T-cell-depleting induction therapy used (alemtuzumab or thymoglobulin). Finally, we reperformed our main analyses after restricting the study population to those who had GN as their native disease (control group restricted to those with GN but received PTI) and also after excluding those who received only prednisone as PTI.

## Results

### Population Characteristics

**Figure 1.**
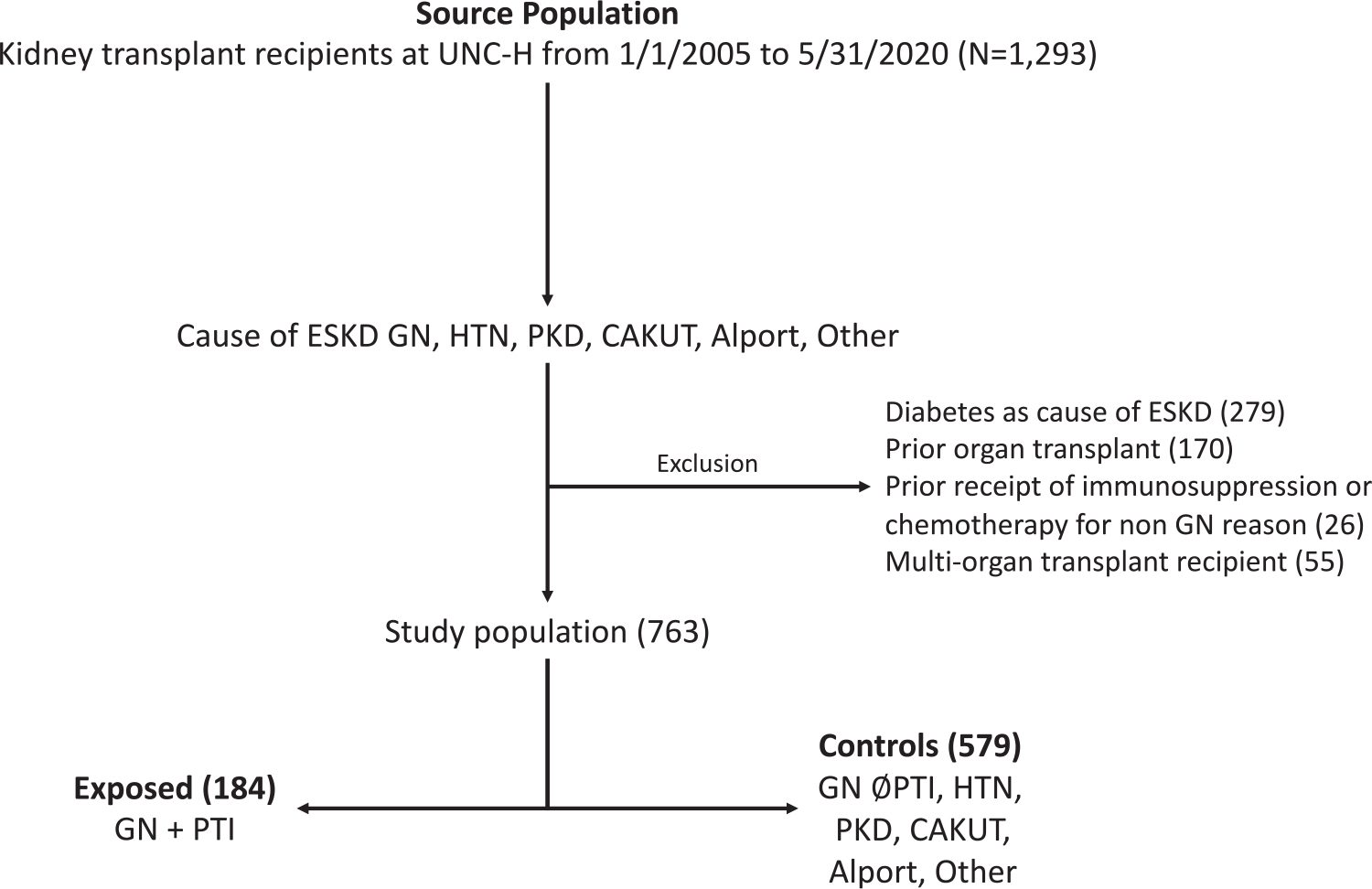
Study flow chart for cohort creation. Abbreviations: CAKUT, congenital anomalies of the kidneys and urinary tracts; ESKD, end-stage kidney disease; GN, glomerulonephritis; HTN, hypertension; PKD, polycystic kidney disease; PTI, pretransplant immunosuppression; UNC-H, University of North Carolina Hospital.

A total of 763 kidney transplant patients were followed for a median 5.7 years. The mean age was 43.2 years, 44.4% were female, 43.9% were White, and 42.9% were Black. Living donor transplant recipients comprised 35.8% of the total cohort, and 85.9% received T-cell-depleting induction, primarily alemtuzumab (86.6%) (Table 1). There were 331 who had GN as a cause of ESKD, of which 127 (38.4%) had FSGS, 55 (16.6%) IgAN, 36 (10.9%) had LN, 33 (10.0%) had ANCA vasculitis, 19 (5.7%) had MN, and 61 (18.4%) had another type of GN. There were 184 who had GN and PTI (of whom 31.0% had FSGS, 13.6% had IgA, 18.0% had LN, 17.4% had ANCA vasculitis, 7.6% had membranous, and 12.5% had another type of GN), and 579 controls (see Fig 1). The patients who had GN and PTI were more likely to be female, were younger, had a shorter dialysis vintage, and were less likely to have early steroid withdrawal compared with the control patients.

### Pretransplant Immunosuppression in Patients With GN

Out of 184 patients with GN who received PTI, 81 (44.0%) had received cyclophosphamide, 31 (16.9%) received rituximab, 83 (45.1%) received MMF, 18 (9.8%) received azathioprine, 60 (32.6%) received CNI, and 170 (92.4%) had a course of high-dose prednisone at some point before transplant for treatment of GN. The median cumulative dose of cyclophosphamide as PTI was 6 g, and for rituximab it was 2 g. The median duration of MMF was 12 months, for azathioprine 17 months, and for CNI 20.5 months (Table 2).

### Viral Infections

**Figure 2.**
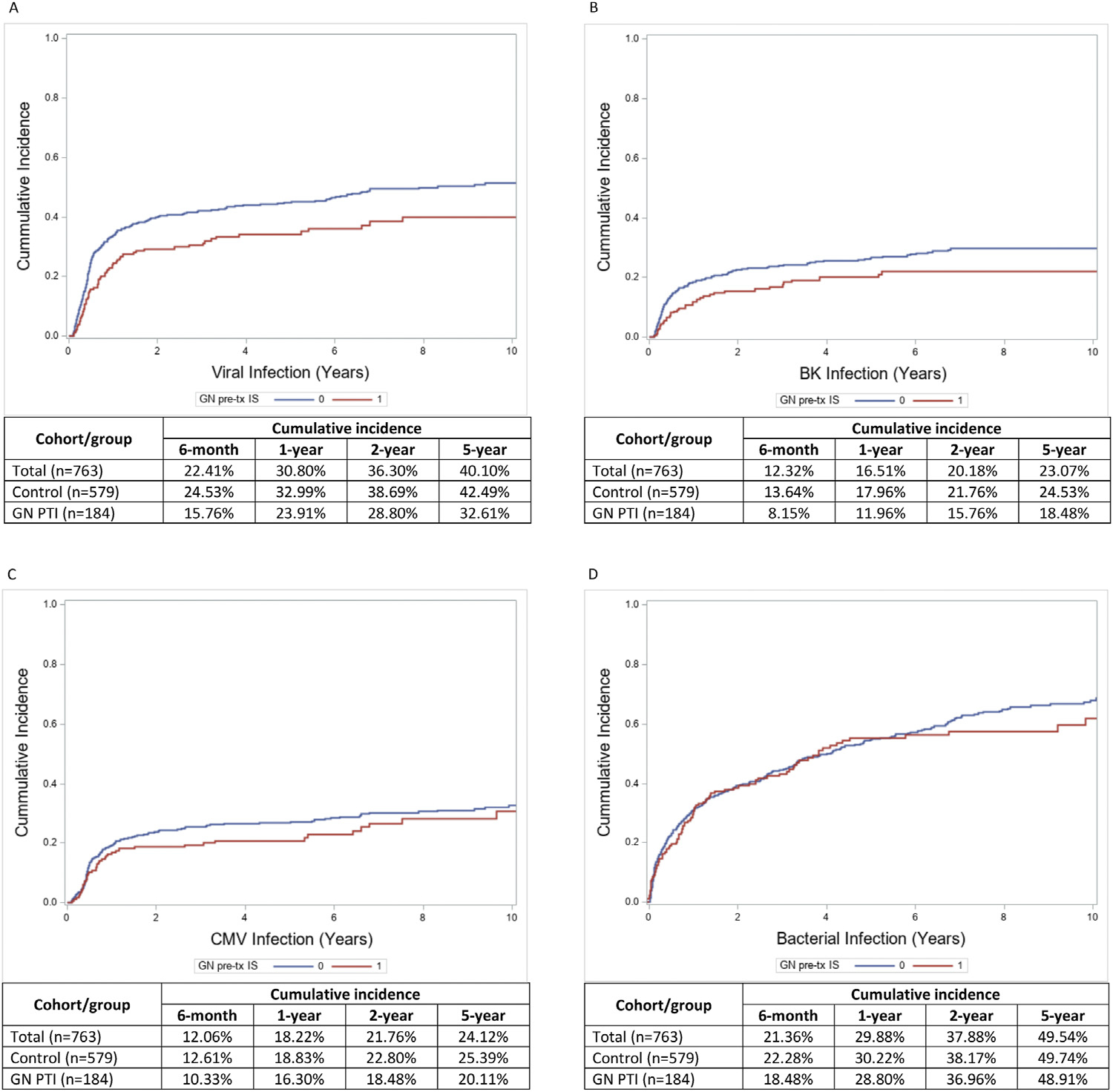
Kaplan-Meier curves and crude cumulative incidences for infections. (A) Kaplan-Meier curves of time to viral infection, with cumulative incidences. (B) Kaplan-Meier curves of time to BK virus infection, with cumulative incidences. (C) Kaplan-Meier curves of time to CMV infection, with cumulative incidences. (D) Kaplan-Meier curves of time to bacterial infection, with cumulative incidences. Abbreviation: CMV, cytomegalovirus; GN, glomerulonephritis; GN PTI, glomerulonephritis having received pretransplant immunosuppression; IS, immunosuppression; tx, transplant.

There were 335 study participants (43.9%) who developed a first viral infection at a median 178 days after transplant: 66 patients (35.9%) in the GN PTI group at a median 243 days, and 269 patients (46.5%) in the control group at a median 175 days. From within 6–12 months after transplant, the cumulative incidence of viral infections was lower in GN PTI compared with the control group and remained as such throughout the study period (Fig 2A–C). The receipt of PTI for GN was not associated with an increased risk for viral infection (AHR, 0.69 [95% CI, 0.52–0.91]). When looking at only BK infection or CMV infection, GN PTI was also not associated with an increased risk for these infections (AHR, 0.72 [95% CI, 0.50–1.05] and 0.76 [95% CI, 0.54–1.07], respectively) (Table 3). The detailed Cox models are presented in Table S1. Participants with GN PTI did not have more severe viral infections compared with controls; they had similar peak BK and CMV viremias; and BK nephropathy was not more common in GN PTI patients (Table 4).

### Bacterial Infections

There were 432 study participants (56.6%) who developed a first bacterial infection at a median 324 days after transplant: 99 patients (53.8%) in the GN PTI group at a median 339 days, and 333 patients (57.5%) in the control group at a median 322 days. The most common types of first bacterial infection were urinary tract infections (62.7%), then pneumonia (13.7%), skin and soft tissue infections (8.6%), and bacteriemia (6.9%) (Table S2). The cumulative incidence of bacterial infection remained fairly similar throughout the study period (Fig 2D). Participants with GN PTI were not at an increased risk for developing bacterial infection compared with the controls (AHR, 0.90 [95% CI, 0.71–1.13]) (Table 3).

### Infection Risks by Type of PTI

There was no specific type of PTI (cyclophosphamide, rituximab, MMF, or CNI) that was associated with an increased risk for developing infection, either viral or bacterial. Higher amounts of cyclophosphamide and rituximab received as PTI were also not associated with an increased risk for infection (Table S3).

### Graft Outcomes

Rejection and de novo donor-specific antibody (DSA) formation occurred at similar rates between GN PTI and controls, as did the rates of graft loss and death (Table S4). Recurrence of GN after transplant was similar between those who received GN PTI and those with GN as native disease who never received PTI (18.5% vs 13.6%, respectively; *P* = 0.29).

### Additional Analyses

When examining participants who developed only a viral infection (and no bacterial infection), only a bacterial infection (and no viral infection), or both a viral and bacterial infection throughout the study period, there was no increased risk associated with the receipt of PTI for GN (Table S5). When stratifying analyses by the type of T-cell-depleting induction therapy used at time of transplant (either alemtuzumab or thymoglobulin), there was no increased risk for any type of infection with GN PTI (Table S6). Restricting the study population to only those who had GN as their native kidney disease, we again found results similar to our main findings (Table S7). There were only 28 individuals who were exposed to only prednisone as PTI; excluding these 28 from the analysis did not yield major differences in outcomes (Table S8).

## Discussion

In this retrospective single-center study examining risk for infection after kidney transplant associated with PTI for treatment of native GN, there was no increased risk for developing a first viral (neither BK nor CMV infection) nor first bacterial infection. We also did not find that a specific type of PTI was associated with an increased risk for infections after transplant. Our findings suggest that PTI for the treatment of GN likely does not contribute significantly to the immunosuppression burden after kidney transplant such as to predispose patients to posttransplant infection.

The impact of PTI on the risks for adverse outcomes after kidney transplant is an understudied area. A few studies have examined the risk for malignancy posttransplant and found that PTI increased the risks for breast cancer, lymphoma, and non-melanoma skin cancer.^7–10^ As it pertains to a comprehensive evaluation of infectious risks after transplant from PTI for the treatment of GN, this had yet to be properly examined. We found that viral and bacterial infections were quite common in our kidney transplant population, but they were not more common among those who had received GN PTI.

Given the known effect of higher doses and longer duration of immunosuppression on the risk for infections in kidney transplant patients,^5,12,13^ our finding that PTI does not increase the risk for infection after transplant, neither viral nor bacterial, is relevant. Indeed, clinicians sometimes reconsider their usual choices for induction therapy or doses of maintenance immunosuppression in patients who have had significant exposure to immunosuppression before transplant. Some reports have suggested using antithymocyte globulin induction therapy may decrease the recurrence of glomerular disease after transplant, in particular IgA nephropathy^14,15^; however, there may be concerns of early infections risk with T-cell-depleting induction agents in a patient who has had extensive PTI exposure. Our study suggests such concerns may not be warranted, even with use of alemtuzumab which is regarded as a more potent, longer lasting T-cell-depleting agent.^11^

Although our finding that PTI does not increase the risk for infections after transplant is relevant, the fact that our results seem to suggest that PTI for the treatment of GN reduces the risk for viral infections was unexpected and contrary to our prespecified hypothesis. Consistently and throughout multiple additional analyses we found that patients with GN PTI had approximately 20%–30% less risk for developing a first viral infection for both BK and CMV infections. Furthermore, the viral infections in GN PTI were not of greater severity: the peak viremia measurements for BK and CMV were similar between the 2 groups, and BK nephropathy also seemed to occur less commonly. Interestingly, this reduction was not seen for bacterial infections where the HR was mostly around 1.

While it is difficult to reconcile how PTI could reduce the risk for viral and not bacterial infection, one consideration is of uncaptured confounding, perhaps in the follow-up of patients with GN PTI or the doses of antimetabolite agents used after transplant. Our patients with GN PTI had baseline characteristics that could suggest an inherently healthier, more adherent population than our controls (younger, lower dialysis vintage). However, such features should impact bacterial infections as well, and we controlled for imbalanced baseline characteristics in our models. Furthermore, adherent patients should have better follow-up observation, which would likely lead to more opportunities to detect BK and CMV infections because these are usually diagnosed in routine posttransplant follow-up.

Our transplant population is evaluated regularly in the first 2 years after transplant, regardless of whether they also visit a community nephrologist closer to home, such that both treatment groups should have had similar opportunities for BK and CMV infection diagnosis. Maintenance immunosuppression dosing in our center also is fairly protocolized for all patients unless they suffer a major complication such as a serious infection, leading to dose adjustments; such adjustments would occur after the infection outcome in our study. Immunologic complications such as rejection and de novo DSA formation could lead to increases in immunosuppression affecting subsequent infection outcomes. However, these did not occur more frequently in one of the groups, and they tended to occur after the occurrence of a first infection.

Another consideration is that PTI could predispose patients to marrow suppression from antimetabolite use after transplant, leading to dose reductions. Again, this should impact the risk for bacterial infections similarly. Because we cannot find a reasonable explanation, either through some unmeasured confounding or through some mechanistic process, this intriguing finding of possibly reduced viral infection risk would need to be confirmed in future studies. However, our primary finding that GN PTI treatment does not seem to increase the risk for adverse infectious complications after kidney transplant remains. A potential reduction in viral infections in PTI patients certainly does not suggest that monitoring for infections should be any different for these patients than any other kidney transplant recipient. Taken together, we would assert that infection surveillance should be similar in any kidney transplant recipient.

The major strength of our study was the use of chart review for confirming exposure and outcomes, thus reducing the risk for misclassification. We also were able to determine the effect of different types of PTI used for the treatment of GN. Our findings are limited in that it is a single-center study where many of our transplant patients with GN PTI would have had their GN treated at UNC. The results may not be generalizable to other centers where GN may be treated differently, with different scale and types of immunosuppression. Furthermore, misclassification is possible because some infections may have occurred outside UNC and hence may not have been captured. This would be unlikely for BK or CMV infection because these are typically diagnosed during a transplant follow-up visit. Although this misclassification could occur for bacterial infections, we would have no reason to believe it would differentially affect the GN PTI group, so it would be unlikely to significantly change our findings.

Due to their uncommon occurrence, we did not capture other opportunistic infections such as mycobacterial, fungal, or *Pneumocystis jiroveci* infections. Our results do not apply to these infections, but we feel it would be unlikely that PTI would increase the risk for these and not viral or bacterial infections. Because the patients exposed to a specific type of PTI could have also been exposed to other types of PTI, it is difficult to isolate the effect of a single type of immunosuppressant on their risk for infections after transplant because of the limited number of patients who were exposed to a single agent. Also, we did not capture methylprednisolone use for treatment of GN before transplant, so our findings cannot establish its potential effect on adverse outcomes after transplant.

Finally, as an observational study, the risk for unmeasured confounding remains. As detailed previously, this may particularly be the case for the noted unexpected trend in lower viral infection risk with GN PTI. For example, we did not consider changes in graft function (serum creatinine, proteinuria) after transplant, which could impact the frequency of follow-up visits and testing for viral infections. Even with an unmeasured confounder, the AHRs for viral infections would most likely be closer to the null. Therefore, our main finding that GN PTI does not seem to be associated with an increased risk for posttransplant infections would likely remain.

In this single-center cohort study of kidney transplant recipients, PTI for treatment of native GN was not associated with an increased risk for developing a first viral or bacterial infection after kidney transplant. While the previously described risks for malignancy must be considered,^10^ patients with glomerular diseases with significant PTI exposure may not require more augmented surveillance for infections after transplant.

## Supplementary Material

1 **Item S1:** Missing duration or dosage of pretransplant immunosuppression.
**Item S2:** UNC kidney transplant immunosuppression protocol.
**Table S1:** Detailed Cox model for infection outcomes.
**Table S2:** Types of first infection events after transplant.
**Table S3:** Infection risks by type of PTI.
**Table S4:** Risks for rejection, de novo DSA formation, graft loss, and death associated with GN PTI.
**Table S5:** Subgroups of infections and risks associated with GN PTI.
**Table S6:** Infection risks by type of T-cell-depleting induction used at transplant.
**Table S7:** Crude HRs for outcomes restricted to study population of transplant recipients with GN as their native kidney disease.
**Table S8:** Outcomes when excluding individuals who were only exposed to prednisone as PTI.

## Figures and Tables

**Table 1.** Baseline Characteristics

Characteristics	Total Population (n = 763)	GN PTI (n = 184)	Control (n = 579)	*P* Value^a^
Sex female	336 (44.4%)	93 (50.5%)	243 (41.6%)	0.05
Pediatric	90 (11.8%)	24 (13.0%)	66 (11.4%)	0.6
Age	46 [31–57]	375 [24.5–52]	49 [35–58]	<0.001
Age category				0.7
<12	43 (5.6%)	11 (6.0%)	32 (5.5%)	
12–17	47 (6.2%)	13 (7.7%)	34 (5.8%)	
18–65	604 (79.2%)	147 (79.9%)	457 (78.9%)	
>65	69 (9.0%)	13 (7.1%)	56 (9.7%)	
Race/ethnicity				
White	335 (43.9%)	89 (48.4%)	246 (42.5%)	0.2
Black	327 (42.9%)	66 (35.8%)	261 (45.1%)	
Hispanic	63 (8.3%)	18 (9.8%)	45 (7.8%)	
Other	38 (5.0%)	11 (6.0%)	27 (4.7%)	
Year of transplant				0.01
2005–2009	222 (29.1%)	38 (20.7%)	184 (31.8%)	
2010–2014	234 (30.7%)	64 (34.8%)	1 70 (29.4%)	
2015–2020	307 (40.2%)	82 (44.6%)	225 (38.9%)	
Native kidney disease				
GN	331 (43.4%)	184 (100%)	147 (25.4%)	
Hypertension	154 (20.2%)	—	154 (26.6%)	
PKD	81 (10.6%)	—	81 (14.0%)	
CAKUT	80 (10.5%)	—	80 (13.8%)	
Unknown/other	117 (15.3%)	—	11 7 (20.2%)	
Dialysis vintage mo	44.7 [17.1–78.3]	29.3 [12.5–69.3]	51.1 [19.9–81.3]	<0.001
Pre-emptive transplant	148 (19.4%)	36 (19.6%)	112(19.3%)	0.9
Donor type				0.04
Living	273 (35.8%)	79 (42.9%)	194 (33.5%)	
DBD	398 (52.2%)	89 (48.4%)	309 (53.4%)	
DCD	92 (12.1%)	16 (8.7%)	76 (13.1%)	
PRA %	6.0 ± 19.1	5.1 ± 17.2	6.3 ± 19.7	0.8
History of diabetes pretransplant	35 (4.6%)	10 (5.4%)	25 (4.3%)	0.6
Smoking history before transplant	232 (30.4%)	44 (23.9%)	188 (32.5%)	0.03
EBV transplant status				0.2
Donor −/Recipient −	19 (2.5%)	8 (4.4%)	11 (1.9%)	
Recipient +	699 (91.6%)	164 (89.1%)	535 (92.4%)	
Donor +/Recipient −	45 (5.9%)	1 2 (6.5%)	33 (5.7%)	
CMV transplant status				0.1
Donor −/Recipient −	160 (21.0%)	48 (26.1%)	112 (19.3%)	
Recipient +	463 (60.7%)	102 (55.4%)	361 (62.4%)	
Donor +/Recipient −	140 (18.4%)	34 (18.5%)	106 (18.3%)	
T-cell-depleting induction therapy^b^	655 (85.9%)	152 (82.6%)	503 (86.9%)	0.2
Alemtuzumab	567 (74.3%)	128 (69.6%)	439 (75.8%)	
Thymoglobulin	88 (11.5%)	24 (13.0%)	64 (11.1%)	
Maintenance at time of transplant				0.2
Tacrolimus	757 (99.2%)	181 (98.4%)	576 (99.5%)	
Other	6 (0.8%)	3 (1.6%)	3 (0.5%)	
Early steroid withdrawal	540 (70.8%)	111 (60.3%)	429 (74.1%)	<0.001
Delayed graft function	169 (22.2%)	29 (15.8%)	140 (24.2%)	0.02

Values are given as number (percentage) for categorical variables and as mean ± SD or median [IQR] for continuous variables.

Abbreviations: CMV, cytomegalovirus; CAKUT, congenital anomalies of the kidney and urinary tract; DBD, donation after brain death; DCD, donation after cardiac death; EBV, Epstein-Barr virus; GN, glomerulonephritis; GN PTI, glomerulonephritis with pretransplant immunosuppression; PKD, polycystic kidney disease; PRA, panel reactive antibodies.

a *P* values were calculated using Fisher exact test for categorical variables and Wilcoxon 2 samples test for continuous variable.

b Thirty-six individuals were imputed the type of depleting induction therapy (31 alemtuzumab and 5 thymoglobulin) when the exact depleting agent was not available through chart review, considering alemtuzumab only started being used in 2006 at our center. There were 108 total participants (14.2%) who received non-T-cell-depleting induction (basiliximab or daclizumab):, 32 (17.4%) in the GN PTI group and 76 (13.1%) in the control group.

**Table 2.** Pretransplant Immunosuppression Use

Immunosuppression	GN PTI (n = 184)
CYC used	81 (44.0%)
0< to <10 g	53 (28.8%)
10 to 25 g	1 7 (9.2%)
>25 g	11 (6.0%)
Cumulative dose, g	6.0 [3.0–13.5]
RTX used	31 (16.9%)
0< to <3	22 (12.0%)
≥3	9 (4.9%)
Cumulative dose, g	2.0 [1.8–3.4]
MMF used	83 (45.1%)
Total duration, mo	12.0 [6.0–470]
AZA used	18 (9.8%)
Total duration, mo	1 7.0 [3.0–30.0]
CNI used	60 (32.6%)
Total duration, mo	20.5 [6.0–41.0]
High-dose prednisone used	1 70 (92.4%)
Days between last dose of PTI and transplant	909 [357–2,392]

Values are given as number (percentage) for categorical variables and as median [IQR] for continuous variables. Percentages do not total 100 because a given participant could have received more than 1 type of immunosuppressant before transplant for the treatment of GN.

Abbreviations: AZA, azathioprine; CNI, calcineurin inhibitor; CYC, cyclophosphamide; GN PTI, glomerulonephritis with pretransplant immunosuppression; MMF, mycophenolate; PTI, pretransplant immunosuppression; RTX, rituximab.

**Table 3.** Infections and Risks Associated With GN PTI

Outcome	Total Population (n = 763)	GN PTI n = 184	Control n = 579
Follow-up, y	5.7 [3.0–9.2]	5.1 [2.9–8.6]	5.8 [3.0–9.7]
Loss-to-follow-up	36 (4.7%)	6 (3.3%)	30 (5.2%)
Viral infection^a^			
N (%)	335 (43.9%)	66 (35.9%)	269 (46.5%)
Rate of first infection per 100 PY	10.1	77	11.0
Days to occurrence	178 [112–436]	243 [121–446]	1 75 [107–430]
Univariate HR (95% CI)		0.70 (0.53–0.91)	Reference
Adjusted HR (95% CI)		0.69 (0.52–0.91)	Reference
BK infection^b^			
N (%)	188 (24.6%)	36 (19.6%)	152 (26.3%)
Rate of first infection per 100 PY	4.8	3.8	5.2
Days to occurrence	184 [96.5–532]	270.5 [117.5–577.5]	171 [92–530]
Univariate HR (95% CI)		0.70 (0.49–1.01)	Reference
Adjusted HR (95% CI)		0.72 (0.50–1.05)	Reference
CMV infection^c^			
N (%)	213 (27.9%)	45 (24.5%)	1 68 (29.0%)
Rate of first infection per 100 PY	5.3	4.8	5.5
Days to occurrence	234 [147–560]	242 [140–551]	213 [149.5–567]
Univariate HR (95% CI)		0.83 (0.60–1.15)	Reference
Adjusted HR (95% CI)		0.76 (0.54–1.07)	Reference
Bacterial infection			
N (%)	432 (56.6%)	99 (53.8%)	333 (57.5%)
Rate of first infection, per 100 PY	15.8	15.5	15.9
Days to occurrence	324 [72–1,109]	339 [72–969]	322 [71–1,137]
Univariate HR (95% CI)		0.93 (0.74–1.17)	Reference
Adjusted HR (95% CI)		0.90 (0.71–1.13)	Reference

Values are given as number (%) for categorical variables or as median [IQR] for continuous variables unless otherwise indicated.

Abbreviations: CMV, cytomegalovirus; GN PTI, glomerulonephritis having received pretransplant immunosuppression; HR, hazard ratio; PY, person-years.

a Defined as BK infection or CMV infection. Adjusted variables: age, sex, race, type of donor, year of transplant, dialysis vintage, T-cell-depletion induction, and CMV status.

b BK infection defined as BK viremia or BK nephropathy; BK viremia defined as one-time viremia above quantifiable level and BK nephropathy defined as histopathology-confirmed disease. Adjusted variables: age, sex, race, type of donor, year of transplant, dialysis vintage, and T-cell-depletion induction.

c CMV infection defined as 1-time viremia above quantifiable level or histopathology-confirmed disease. Adjusted variables: age, sex, race, type of donor, year of transplant, dialysis vintage, T-cell-depletion induction, and CMV status.

**Table 4.** Severity of Viral Infections

	Total Population (n = 763)	GN PTI (n = 184)	Control (n = 579)	*P* Value
BK viremia	185 (24%)	36 (19.6%)	149 (25.7%)	
Highest BK viremia	12,465.5 [1,862–62,165]	10,701.5 [1,346–50,678.5]	13,606.5 [2,051–62,565]	0.7
CMV viremia	212 (27.8%)	45 (24.5%)	1 67 (28.8%)	
Highest CMV viremia	810 [255.5–3,432.5]	796 [200–2,798]	812 [293–5,476]	0.2
BK nephropathy^a^				
N (%)	74 (9.7%)	10 (5.4%)	64 (11.1%)	
Days to occurrence	177.5 [105–360]	278 [99–541]	167 [105–334]	
Univariate HR		0.47 (0.24–0.91)	Reference	
Adjusted HR		0.65 (0.33–1.30)	Reference	

Values are given as HR (95% CI), number (%) for categorical variables, or as median [IQR] for continuous variables.

Abbreviations: CMV, cytomegalovirus; GN PTI, glomerulonephritis with pretransplant immunosuppression; HR, hazard ratio.

a BK nephropathy defined as histopathologic-confirmed disease.

## Data Availability

Deidentified individual data that supports the results will be shared beginning 9 to 36 months following publication provided the investigator who proposes to use the data has approval from an institutional review board, independent ethics committee, or research ethics board, as applicable, and executes a data use/sharing agreement with UNC.

## PLAIN-LANGUAGE SUMMARY

Many kidney transplant patients have glomerular disease as the cause of kidney failure. These patients may be exposed to immunosuppression before transplantation, which could increase the risk for infections after receipt of a transplanted kidney. We identified kidney transplant recipients at a university teaching hospital who received immunosuppression before transplant for the treatment of glomerular kidney disease. We examined their risk for infection after transplantation by comparing it with the risk among transplant patients who were not exposed to immunosuppression before transplant. We observed no increased risk for infection after exposure to prior immunosuppression. Therefore, patients exposed to significant amounts of immunosuppression before transplantation may not require special surveillance or medication adjustment for fear of infection after their receipt of a kidney transplant.

## References

[1] Chan S, Pascoe EM, Clayton PA, Infection-related mortality in recipients of a kidney transplant in Australia and New Zealand. Clin J Am Soc Nephrol. 2019;14(10):1484–1492. doi:10.2215/CJN.03200319 31455690 PMC6777595

[2] Cowan J, Bennett A, Fergusson N, Incidence rate of post-kidney transplant infection: a retrospective cohort study examining infection rates at a large Canadian multicenter tertiary-care facility. Can J kidney Heal Dis. 2018;5:2054358118799692. doi:10.1177/2054358118799692 PMC6136109 30224973

[3] Snyder JJ, Israni AK, Peng Y, Zhang L, Simon TA, Kasiske BL. Rates of first infection following kidney transplant in the United States. Kidney Int. 2009;75(3):317–326. doi:10.1038/ki.2008.580 19020531

[4] San Juan R, Aguado JM, Lumbreras C, Impact of current transplantation management on the development of cytomegalovirus disease after renal transplantation. Clin Infect Dis. 2008;47(7):875–882. doi:10.1086/591532 18752439

[5] Dadhania D, Snopkowski C, Ding R, Epidemiology of BK virus in renal allograft recipients: independent risk factors for BK virus replication. Transplantation. 2008;86(4):521–528. doi:10.1097/TP.0b013e31817c6447 18724220 PMC3647687

[6] Lentine KL, Smith JM, Hart A, OPTN/SRTR 2020 annual data report: kidney. Am J Transplant. 2022;22(suppl 2):21–136. doi:10.1111/AJT.16982 35266618

[7] Jorgenson MR, Descourouez JL, Singh T, Astor BC, Panzer SE. Malignancy in renal transplant recipients exposed to cyclophosphamide prior to transplantation for the treatment of native glomerular disease. Pharmacotherapy. 2018;38(1):51–57. doi:10.1002/phar.2059 29136299

[8] Hibberd AD, Trevillian PR, Wlodarzcyk JH, Predialysis immunosuppression is an independent risk factor for some cancers in renal transplantation. Transplant Proc. 2001;33(1–2):1846–1847. doi:10.1016/S0041-1345(00)02704-4 11267538

[9] Hibberd AD, Trevillian PR, Wlodarczyk JH, Effect of immunosuppression for primary renal disease on the risk of cancer in subsequent renal transplantation: a population-based retrospective cohort study. Transplantation. 2013;95(1):122–127. doi:10.1097/TP.0b013e3182782f59 23238532

[10] Massicotte-Azarniouch D, Detwiler RK, Hu Y, Malignancy risk in kidney transplant recipients exposed to immunosuppression pre-transplant for the treatment of glomerulonephritis. Nephrol Dial Transplant. 2022;16(3):518–524. doi:10.1093/NDT/GFAC337 PMC10468752 36549661

[11] Hanaway MJ, Woodle ES, Mulgaonkar S, Alemtuzumab induction in renal transplantation. N Engl J Med. 2011;364(20):1909–1919. doi:10.1056/nejmoa1009546 21591943

[12] Hartmann A, Sagedal S, Hjelmesæth J. The natural course of cytomegalovirus infection and disease in renal transplant recipients. Transplantation. 2006;82:S15–S17. doi:10.1097/01.tp.0000230460.42558.b0 16858268

[13] Höcker B, Schneble L, Murer L, Epidemiology of and risk factors for BK polyomavirus replication and nephropathy in pediatric renal transplant recipients: an international CERTAIN registry study. Transplantation. 2019;103(6):1224–1233. doi:10.1097/TP.0000000000002414 30130322

[14] Berthoux F, El Deeb S, Mariat C, Diconne E, Laurent B, Thibaudin L. Antithymocyte globulin (ATG) induction therapy and disease recurrence in renal transplant recipients with primary iga nephropathy. Transplantation. 2008;85(10):1505–1507. doi:10.1097/TP.0b013e3181705ad4 18497694

[15] Pascual J, Mezrich JD, Djamali A, Alemtuzumab induction and recurrence of glomerular disease after kidney transplantation. Transplantation. 2007;83(11):1429–1434. doi:10.1097/01.tp.0000264554.39645.74 17565315

