# Supplementary material for: Infections Following Kidney Transplantation After Exposure to Immunosuppression for Treatment of Glomerulonephritis
Source: Am J Kidney Dis. Author manuscript; Available in PMC 2024 Dec 20. (PMC11660976; doi:10.1053/j.ajkd.2023.10.016)
Supplement: 1 — Item S1: Missing duration or dosage of pretransplant immunosuppression. Item S2: UNC kidney transplant immunosuppression protocol. Table S1: Detailed Cox model for infection outcomes. Table S2: Types of first infection events after transplant. Table S3: Infection risks by type of PTI. Table S4: Risks for rejection, de novo DSA formation, graft loss, and death associated with GN PTI. Table S5: Subgroups of infections and risks associated with GN PTI. Table S6: Infection risks by type of T-cell-depleting induction used at transplant. Table S7: Crude HRs for outcomes restricted to study population of transplant recipients with GN as their native kidney disease. Table S8: Outcomes when excluding individuals who were only exposed to prednisone as PTI. [file NIHMS2037196-supplement-1.pdf]

**Item S1.** Missing duration or dosage of pre-transplant immunosuppression

Prednisone use (dosage and duration) was difficult to ascertain through chart review because of inconsistent documentation of the exact dose in the notes. Therefore, we did not attempt to quantify this exposure in our study. For the other immunosuppressant use, there were 20 instances when the type of PTI received was known but the exact information on the dosage or duration was not available in the chart (for example, a patient with ANCA vasculitis received induction therapy with cyclophosphamide IV, but no information on the number or dosage of infusions). We had to impute these values using estimates that were on the conservative side. For cyclophosphamide, we imputed 0.8g for each IV dose given (based on 0.5g/$m^2$ for an average of 1.7$m^2$ body surface area) and 75mg/day when oral was given. When the duration was not known, we imputed 3 months (so 3 IV doses or 90 days of oral therapy). For rituximab, an IV dose was imputed as 0.6g when it was a weekly x 4 regimen (based on 375mg/$m^2$ for an average of 1.7$m^2$ body surface area), and it was imputed 1g when it was a 2 doses 14 days apart dosing regimen. When the duration of mycophenolate, azathioprine or calcineurin inhibitor use were not known, a 6-month course of treatment was imputed.

**Item S2.** UNC kidney transplant immunosuppression protocol

The following are the general guidelines for use of immunosuppression in kidney transplant recipients at UNC from our 2021 updated guidelines. Although the study period spans from 2005 until 2020 and the guidelines may have changed somewhat throughout this period, the major points of monitoring and treatment have remained fairly similar. Differences may exist in individual patients based on clinical judgement of the treating clinician.

Patients usually receive alemtuzumab at time of transplant as induction therapy along with 4 days of solumedrol. When alemtuzumab is used, patients typically have early steroid withdrawal with no further corticosteroids after the induction solumedrol. Maintenance therapy consists of tacrolimus as the drug of choice, with a target trough 8-10 ng/ml in the first 3 months, then 6-8 ng/ml from 4-12 months, then as per treating physician discretion. The anti-metabolite of choice is mycophenolate mofetil at 750mg BID (or equivalent mycophenolic acid). Those who do not receive alemtuzumab induction typically will not have early steroid withdrawal and will be kept on 5mg prednisone long-term. For example, patients on chronic prednisone prior to transplant and those who receive an HCV positive kidney will receive induction with ATG and will be kept on 5mg prednisone maintenance therapy. Patients of older age (>70 years old) with PRA 0% typically receive basiliximab induction and will not have early steroid withdrawal either. Protocol allograft biopsies are not performed. Donor specific antibody (DSA) screening is typically done as surveillance 4 times in the first-year post-transplant then 2-4 times per year until year 3, then yearly. General guidelines for the management of rejection at our institution are as follows: acute cellular rejection is treated with 3 days of methylprednisolone pulse IV then oral prednisone taper, with thymoglobulin

reserved for cases of severe rejection or steroid-resistant rejection; antibody mediated rejection is treated with IVIg, therapeutic plasma exchange with or without rituximab. There are no specific management guidelines for de novo DSA formation however, patients may be kept on higher doses of chronic immunosuppression and there may be a lower threshold to perform a kidney biopsy.

Viral prophylaxis is used according to transplant CMV risk status. High risk transplants (donor +/recipient -) receive valganciclovir for 6 months; moderate risk transplants (recipient +) receive valganciclovir for 3 months; low risk transplants (donor -/recipient -) receive oral acyclovir for 3 months. Routine monitoring for CMV viremia is not done except for high-risk transplants where weekly CMV PCR for 8 weeks is done upon completion of the 6 months of prophylactic valganciclovir. All patients receive pneumocystis jiroveci pneumonia prophylaxis for 6 months post-transplant, most commonly with trimethoprim-sulfamethoxazole. Screening for polyoma virus infection is done with urine cytology decoy cell testing at weeks 2 and 4, then at months 3, 6, 9, 12, 15, 18, 24 and 36. If there are >10 decoy cells/HPF, this prompts BK DNA PCR serum testing where, based on the result of the PCR, either a kidney biopsy will be performed to rule out polyoma virus associated nephropathy (for higher PCR levels), the PCR level will simply be monitored (for lower PCR levels) or there will be immediate reduction in immunosuppression.

**Table S1.** Detailed cox model for infection outcomes

| Outcomes | variables | | Estimate | HR (95%CI) | P value |
|---|---|---|---|---|---|
| **Viral infection** | gn_pti | Case vs. Control | -0.3706 | 0.69(0.52, 0.91) | 0.0085 |
| | age_tx | | 0.0029 | 1.00(1.00, 1.01) | 0.4281 |
| | sex | Male vs. Female | 0.0399 | 1.04(0.84, 1.30) | 0.7206 |
| | race | black vs. white | 0.2914 | 1.34(1.02, 1.76) | 0.0369 |
| | race | Hispanic vs. white | 0.1193 | 1.13(0.72, 1.76) | 0.5987 |
| | race | Other vs. white | 0.5824 | 1.79(1.12, 2.86) | 0.0148 |
| | donor | Brain death vs. Living | 0.0742 | 1.08(0.81, 1.43) | 0.6081 |
| | donor | Cardiac death vs. Living | 0.2362 | 1.27(0.86, 1.86) | 0.2291 |
| | txyear | | 0.0899 | 1.09(1.06, 1.12) | <0.0001 |
| | vintage_year | | 0.0099 | 1.01(0.98, 1.05) | 0.5733 |
| | depletion_ind | Yes vs. No | -0.1909 | 0.83(0.60, 1.14) | 0.2511 |
| | cmvstatus | R+ vs D-/R- | 0.4543 | 1.58(1.14, 2.18) | 0.0061 |
| | cmvstatus | D+/R- vs D-/R- | 0.5843 | 1.79(1.24, 2.60) | 0.002 |
| **BK infection** | gn_pti | Case vs. Control | -0.3271 | 0.72(0.50, 1.05) | 0.0848 |
| | age_tx | | 0.0058 | 1.01(1.00, 1.01) | 0.2052 |
| | sex | Male vs. Female | 0.265 | 1.30(0.97, 1.76) | 0.0809 |
| | race | black vs. white | 0.1423 | 1.15(0.80, 1.66) | 0.4407 |
| | race | Hispanic vs. white | 0.4115 | 1.51(0.88, 2.59) | 0.1343 |
| | race | Other vs. white | 0.5412 | 1.72(0.94, 3.14) | 0.0789 |
| | donor | Brain death vs. Living | 0.035 | 1.04(0.71, 1.51) | 0.8549 |
| | donor | Cardiac death vs. Living | 0.0271 | 1.03(0.61, 1.72) | 0.9182 |
| | txyear | | 0.0375 | 1.04(1.00, 1.07) | 0.0325 |
| | vintage_year | | 0.0252 | 1.03(0.98, 1.07) | 0.2761 |
| | depletion_ind | Yes vs. No | -0.3824 | 0.68(0.46, 1.02) | 0.0621 |
| **BK nephropathy** | gn_pti | Case vs. Control | -0.4246 | 0.65(0.33, 1.30) | 0.2237 |
| | age_tx | | 0.0259 | 1.03(1.01, 1.04) | 0.002 |
| | sex | Male vs. Female | 0.4583 | 1.58(0.97, 2.58) | 0.0659 |
| | race | black vs. white | 0.392 | 1.48(0.83, 2.65) | 0.1869 |
| | race | Hispanic vs. white | 0.2399 | 1.27(0.46, 3.52) | 0.6445 |
| | race | Other vs. white | 0.6848 | 1.98(0.74, 5.35) | 0.1763 |
| | donor | Brain death vs. Living | 0.0239 | 1.02(0.54, 1.95) | 0.942 |
| | donor | Cardiac death vs. Living | 0.1962 | 1.22(0.55, 2.68) | 0.6266 |
| | txyear | | -0.0294 | 0.97(0.92, 1.02) | 0.281 |
| | vintage_year | | 0.0521 | 1.05(0.98, 1.13) | 0.152 |
| | depletion_ind | Yes vs. No | 0.0953 | 1.10(0.52, 2.34) | 0.8044 |
| **CMV infection** | gn_pti | Case vs. Control | -0.269 | 0.76(0.54, 1.07) | 0.1206 |
| | age_tx | | -0.0038 | 1.00(0.99, 1.01) | 0.4008 |
| | sex | Male vs. Female | -0.1688 | 0.84(0.64, 1.11) | 0.2259 |
| | race | black vs. white | 0.3473 | 1.42(1.00, 2.00) | 0.0491 |

| | | | | | |
|---|---|---|---|---|---|
| | race | Hispanic vs. white | 0.0878 | 1.09(0.64, 1.88) | 0.7509 |
| | race | Other vs. white | 0.4834 | 1.62(0.92, 2.86) | 0.094 |
| | donor | Brain death vs. Living | 0.0387 | 1.04(0.73, 1.48) | 0.8314 |
| | donor | Cardiac death vs. Living | 0.3009 | 1.35(0.84, 2.18) | 0.2157 |
| | txyear | | 0.132 | 1.14(1.10, 1.18) | <0.0001 |
| | vintage_year | | -0.0148 | 0.99(0.94, 1.03) | 0.5085 |
| | depletion_ind | Yes vs. No | -0.1025 | 0.90(0.60, 1.37) | 0.6279 |
| | cmvstatus | R+ vs D-/R- | 1.5484 | 4.70(2.63, 8.41) | <0.0001 |
| | cmvstatus | D+/R- vs D-/R- | 1.5423 | 4.68(2.51, 8.70) | <0.0001 |
| **Bacterial infection** | gn_pti | Case vs. Control | -0.1094 | 0.90(0.71, 1.13) | 0.3567 |
| | age_tx | | -0.0012 | 1.00(0.99, 1.00) | 0.6937 |
| | sex | Male vs. Female | -0.7147 | 0.49(0.40, 0.60) | 0 |
| | race | black vs. white | -0.079 | 0.92(0.73, 1.17) | 0.5175 |
| | race | Hispanic vs. white | 0.0754 | 1.08(0.73, 1.58) | 0.7009 |
| | race | Other vs. white | 0.1751 | 1.19(0.76, 1.87) | 0.4488 |
| | donor | Brain death vs. Living | 0.1005 | 1.11(0.87, 1.41) | 0.4176 |
| | donor | Cardiac death vs. Living | 0.0329 | 1.03(0.73, 1.47) | 0.8536 |
| | txyear | | 0.0001 | 1.00(0.98, 1.02) | 0.9964 |
| | vintage_year | | 0.0453 | 1.05(1.01, 1.08) | 0.0065 |
| | depletion_ind | Yes vs. No | -0.2815 | 0.75(0.58, 0.99) | 0.0414 |

**Table S2.** Types of first infection events post-transplant

| | **Number (% of total)** |
|---|---|
| **Total** | **433** |
| Urinary tract infection (UTI, urosepsis, prostatitis, scrotal abscess) | 272 (62.8) |
| Pneumonia | 59 (13.6) |
| Skin and soft tissue infection (cellulitis, skin abscess, osteomyelitis, septic arthritis) | 37 (8.5) |
| Bacteremia | 30 (6.9) |
| Intra-abdominal infections (peritonitis, appendicitis, diverticulitis, enteritis, *Clostridioides difficile* colitis, abscess) | 21 (4.8) |
| Sepsis of unclear source | 5 (1.2) |
| Other: sinusitis [2], acute otitis media [1], meningitis [1], tuberculosis [2], syphilis [1], strongiloïdes [1], aspergillosis [1] | 9 (2.1) |

**Table S3.** Infection risks by type of PTI
No signal that one type of PTI is better or worse in terms of infection risks

| PTI (N) | Viral infection | | BK infection | | CMV infection | | Bacterial infection | |
|---|---|---|---|---|---|---|---|---|
| | N (%) | aHR (95%CI)[1] | N (%) | aHR (95%CI)[2] | N (%) | aHR (95%CI)[1] | N (%) | aHR (95%CI)[2] |
| **CYC** | | | | | | | | |
| Controls (579) | 269 (46.5%) | Reference | 152 (26.3%) | Reference | 168 (29.0%) | Reference | 333 (57.5%) | Reference |
| CYC PTI (81) | 32 (39.5%) | 0.76 (0.52-1.10) | 19 (23.5%) | 0.83 (0.51-1.34) | 20 (24.7%) | 0.80 (0.50-1.27) | 49 (60.5%) | 1.04 (0.76-1.40) |
| Controls (579) | 269 (46.5%) | Reference | 152 (26.3%) | Reference | 168 (29.0%) | Reference | 333 (57.5%) | Reference |
| 0< to <10g (53)† | 20 (37.7%) | 0.78 (0.49-1.22) | 12 (22.6%) | 0.86 (0.48-1.54) | 13 (24.5%) | 0.86 (0.49-1.52) | 36 (67.9%) | 1.43 (1.01-2.01) |
| 10 to 25g (17)† | 9 (52.9%) | 0.98 (0.51-1.91) | 4 (23.5%) | 0.80 (0.29-2.15) | 6 (35.2%) | 1.12 (0.49-2.52) | 10 (58.8%) | 1.00 (0.53-1.88) |
| >25g (11)† | 3 (27.3%) | 0.48 (0.15-1.49) | 3 (27.3%) | 0.96 (0.31-3.01) | 1 (9.1%) | 0.27 (0.04-1.94) | 3 (27.3%) | 0.33 (0.11-1.03) |
| **RTX** | | | | | | | | |
| Controls (579) | 269 (46.5%) | Reference | 152 (26.3%) | Reference | 168 (29.0%) | Reference | 333 (57.5%) | Reference |
| RTX PTI (31) | 18 (30%) | 1.17 (0.66-2.09) | 7 (22.6%) | 0.92 (0.42-2.00) | 12 (38.7%) | 1.31 (0.71-2.40) | 19 (61.3%) | 1.40 (0.86-2.26) |
| 0g (579) | 269 (46.5%) | Reference | 152 (26.3%) | Reference | 168 (29.0%) | Reference | 333 (57.5%) | Reference |
| 0< to <3g (22)† | 11 (50.0%) | 1.07 (0.59-1.96) | 5 (22.7%) | 0.85 (0.35-2.06) | 11 (50.0%) | 1.89 (1.02-3.47) | 16 (72.7%) | 1.73 (1.04-2.85) |
| ≥3g (9)† | 2 (22.2%) | 0.47 (0.12-1.87) | 2 (22.2%) | 0.92 (0.23-3.71) | 1 (11.1%) | 0.38 (0.05-2.73) | 3 (33.3%) | 0.61 (0.19-1.89) |
| **MMF** | | | | | | | | |
| Controls (579) | 269 (46.5%) | Reference | 152 (26.3%) | Reference | 168 (29.0%) | Reference | 333 (57.5%) | Reference |
| MMF PTI (83) | 28 (33.7%) | 0.69 (0.46-1.02) | 14 (16.9%) | 0.68 (0.39-1.20) | 22 (26.5%) | 0.84 (0.53-1.34) | 53 (63.9%) | 1.16 (0.85-1.58) |
| **CNI** | | | | | | | | |
| Controls (579) | 269 (46.5%) | Reference | 152 (26.3%) | Reference | 168 (29.0%) | Reference | 333 (57.5%) | Reference |
| CNI PTI (60) | 18 (30.0%) | 0.54 (0.33-0.89) | 9 (15.0%) | 0.53 (0.27-1.06) | 14 (23.3%) | 0.68 (0.39-1.20) | 30 (50.0%) | 0.83 (0.56-1.24) |

1. Adjusted variables: age, sex, race, type of donor, year of transplant, dialysis vintage, T-cell depletion induction and CMV status
2. Adjusted variables: age, sex, race, type of donor, year of transplant, dialysis vintage, T-cell depletion induction

Abbreviations: GN PTI = glomerulonephritis having received pre-transplant immunosuppression, CNI = calcineurin inhibitor, CYC = cyclophosphamide, MMF = mycophenolate, RTX = rituximab
† HRs are crude, unadjusted due to small sample size of the sub-groups and small number of events

**Table S4.** Risks for rejection, de novo DSA formation, graft loss and death associated with GN PTI

Rejection was defined as any biopsy-proven case of acute T-cell mediated rejection, acute antibody mediated rejection, or mixed rejection (both active cellular and antibody mediated rejection present on biopsy). De novo DSA was defined as an HLA tissue typing test demonstrating the presence of an anti-HLA antibody, at any MFI level, in the recipient against the donor which was not present prior to transplant.

| | **Total population (n=763)** | **GN PTI (n=184)** | **Control (n=579)** |
|---|---|---|---|
| **Rejection** | | | |
| N (%) | 217 (28.4%) | 56 (30.4%) | 161 (27.8%) |
| Days to occurrence, median (IQR) | 592 (123.5-1,384.5) | 622 (116-1,256) | 590 (127-1,385) |
| Univariate HR (95%CI) | | 1.18 (0.87-1.60) | Reference |
| Adjusted HR (95%CI) | | 1.12 (0.82-1.53) | Reference |
| **dnDSA** | | | |
| N (%) | 246 (32.2%) | 61 (33.2%) | 185 (32.0%) |
| Days to occurrence, median (IQR) | 547 (100-1,493) | 358 (100-1,135) | 598 (101-1,561) |
| Univariate HR (95%CI) | | 1.11 (0.83-1.48) | Reference |
| Adjusted HR (95%CI) | | 1.09 (0.80-1.47) | Reference |
| **Graft loss** | | | |
| N (%) | 127 (16.6%) | 30 (16.3%) | 97 (16.8%) |
| Days to occurrence, median (IQR) | 1,871 (996-2,784) | 1,691 (1,054-2,402) | 1,947 (996-3,074) |
| Univariate HR (95%CI) | | 1.12 (0.74-1.69) | Reference |
| Adjusted HR (95%CI) | | 1.12 (0.73-1.72) | Reference |
| **Death** | | | |
| N (%) | 92 (12.1%) | 20 (10.9%) | 72 (12.4%) |
| Days to occurrence, median (IQR) | 2,422 (1,022-3356) | 2,051 (722-3,060) | 2,512 (1,064-3,463) |
| Univariate HR (95%CI) | | 1.23 (0.75-2.04) | Reference |
| Adjusted HR (95%CI) | | 1.42 (0.79-2.56) | Reference |

Abbreviations: dnDSA = de novo donor specific antibody, GN PTI = glomerulonephritis having received pre-transplant immunosuppression

**Table S5.** Sub-groups of infections and risks associated with GN PTI

| Outcome | Total population (n=763) | GN PTI (n=184) | Control (n=579) |
|---|---|---|---|
| Viral infection only[1] | | | |
| N (%) | 128 (16.8%) | 26 (20.3%) | 102 (79.7%) |
| Univariate HR (95%CI) | | 0.78 (0.51-1.20) | Reference |
| Adjusted HR (95%CI) | | 0.79 (0.51-1.23) | Reference |
| Bacterial infection only[2] | | | |
| N (%) | 226 (29.6%) | 60 (26.6%) | 166 (73.4%) |
| Univariate HR (95%CI) | | 1.19 (0.88-1.60) | Reference |
| Adjusted HR (95%CI) | | 1.21 (0.89-1.64) | Reference |
| Viral infection & bacterial infection[3] | | | |
| N (%) | 207 (27.8%) | 40 (19.3%) | 167 (80.7%) |
| Univariate HR (95%CI) | | 0.69 (0.49-0.98) | Reference |
| Adjusted HR (95%CI) | | 0.68 (0.48-0.98) | Reference |

1. Individuals who had a viral infection but never had a bacterial infection during the study period. Adjusted variables: age, sex, race, type of donor, year of transplant, dialysis vintage, T-cell depletion induction, cmv status
2. Individuals who had a bacterial infection but never had a viral infection during the study period. Adjusted variables: age, sex, race, type of donor, year of transplant, dialysis vintage, type of induction
3. Individuals who had a viral infection and also had a bacterial infection during the study period. Adjusted variables: age, sex, race, type of donor, year of transplant, dialysis vintage, T-cell depletion induction, cmv status

Abbreviations: GN PTI = glomerulonephritis having received pre-transplant immunosuppression

**Table S6**. Infection risks by type of T-cell depleting induction used at transplant

| | **Alemtuzumab induction (N=567)** | | | **Thymoglobulin induction (N=88)** | | |
|---|---|---|---|---|---|---|
| | **GN PTI (N=128)** | **Control (N=439)** | **Unadjusted HR (95%CI) GN PTI vs control** | **GN PTI (N=24)** | **Control (N=64)** | **Unadjusted HR (95%CI) GN PTI vs control** |
| | **N (%)** | **N (%)** | | **N (%)** | **N (%)** | |
| Viral infection | 50 (39.1) | 202 (46.0) | 0.80 (0.59-1.09) | 6 (25.0) | 30 (46.9) | 0.44 (0.18-1.06) |
| BK infection | 26 (20.3) | 112 (25.5) | 0.77 (0.50-1.17) | 4 (16.7) | 14 (21.9) | 0.70 (0.23-2.11) |
| BK nephropathy | 8 (6.3) | 50 (11.4) | 0.53 (0.25-1.12) | 1 (4.2) | 7 (10.9) | 0.36 (0.04-2.91) |
| CMV infection | 35 (27.3) | 124 (28.2) | 0.98 (0.67-1.43) | 4 (16.7) | 21 (32.8) | 0.46 (0.16-1.34) |
| Bacterial infection | 70 (54.7) | 241 (54.9) | 1.03 (0.79-1.35) | 14 (58.3) | 39 (60.9) | 1.01 (0.55-1.87) |

BK infection, BK nephropathy, Bacterial infection: adjusted for age, sex, race, type of donor, year of transplant, dialysis vintage

Viral infection and CMV infection: adjusted for age, sex, race, type of donor, year of transplant, dialysis vintage, CMV status

Abbreviations: GN PTI = glomerulonephritis having received pre-transplant immunosuppression

**Table S7.** Crude HRs for outcomes restricted to study population of transplant recipients with GN as their native kidney disease.

| | Viral infection | | BK infection | | CMV infection | | Bacterial infection | |
|---|---|---|---|---|---|---|---|---|
| | **Events (%)** | **HR (95%CI)** | **Events (%)** | **HR (95%CI)** | **Events (%)** | **HR (95%CI)** | **Events (%)** | **HR (95%CI)** |
| GN PTI (n=184) | 66 (36.9) | 0.68 (0.48-0.95) | 36 (19.6) | 0.64 (0.41-1.00) | 45 (24.5) | 0.82 (0.54-1.25) | 100 (54.3) | 1.26 (0.93-1.71) |
| GN no PTI (n=147) | 70 (47.6) | Ref | 41 (27.9) | Ref | 44 (29.9) | Ref | 72 (49.0) | Ref |

HRs are unadjusted
Abbreviations: GN PTI = glomerulonephritis having received pre-transplant immunosuppression

**Table S8.** Outcomes when excluding individuals who were only exposed to prednisone as PTI

| | Total population (n=735) | GN PTI (n=156) | Control (n=579) |
|---|---|---|---|
| **Viral infection** | | | |
| N (%) | 323 (44.0) | 54 (34.6) | 269 (46.5) |
| Univariate HR (95%CI) | | 0.68 (0.50-0.90) | Reference |
| Adjusted HR (95%CI) | | 0.67 (0.50-0.90) | Reference |
| **BK infection** | | | |
| N (%) | 183 (24.9) | 31 (19.9) | 152 (26.3) |
| Univariate HR (95%CI) | | 0.72 (0.49-1.06) | Reference |
| Adjusted HR (95%CI) | | 0.74 (0.50-1.10) | Reference |
| **CMV infection** | | | |
| N (%) | 205 (27.9) | 37 (23.7) | 168 (29.0) |
| Univariate HR (95%CI) | | 0.80 (0.56-1.15) | Reference |
| Adjusted HR (95%CI) | | 0.74 (0.51-1.06) | Reference |
| **Bacterial infection** | | | |
| N (%) | 419 (57.0) | 86 (55.1) | 333 (57.5) |
| Univariate HR (95%CI) | | 0.96 (0.76-1.23) | Reference |
| Adjusted HR (95%CI) | | 0.95 (0.74-1.21) | Reference |

Abbreviations: GN PTI = glomerulonephritis having received pre-transplant immunosuppression
